# Modeling Wnt/β-Catenin Target Gene Expression in APC and Wnt Gradients Under Wild Type and Mutant Conditions

**DOI:** 10.3389/fphys.2013.00021

**Published:** 2013-02-25

**Authors:** Uwe Benary, Bente Kofahl, Andreas Hecht, Jana Wolf

**Affiliations:** ^1^Mathematical Modelling of Cellular Processes, Max Delbrück Center for Molecular Medicine Berlin-Buch Berlin, Germany; ^2^Institute of Molecular Medicine and Cell Research, BIOSS Centre for Biological Signalling Studies, Albert-Ludwigs-Universität Freiburg Freiburg, Germany

**Keywords:** mathematical modeling, gradients, gene expression, Wnt/β-catenin signaling, liver, zonation, mutation, feedback

## Abstract

The Wnt/β-catenin pathway is involved in the regulation of a multitude of physiological processes by controlling the differential expression of target genes. In certain tissues such as the adult liver, the Wnt/β-catenin pathway can attain different levels of activity due to gradients of Wnt ligands and/or intracellular pathway components like APC. How graded pathway activity is converted into regionally distinct patterns of Wnt/β-catenin target gene expression is largely unknown. Here, we apply a mathematical modeling approach to investigate the impact of different regulatory mechanisms on target gene expression within Wnt or APC concentration gradients. We develop a minimal model of Wnt/β-catenin signal transduction and combine it with various mechanisms of target gene regulation. In particular, the effects of activation, inhibition, and an incoherent feedforward loop (iFFL) are compared. To specify activation kinetics, we analyze experimental data that quantify the response of β-catenin/TCF reporter constructs to different Wnt concentrations, and demonstrate that the induction of these constructs occurs in a cooperative manner with Hill coefficients between 2 and 5. In summary, our study shows that the combination of specific gene regulatory mechanisms with a time-independent gradient of Wnt or APC is sufficient to generate distinct target gene expression patterns as have been experimentally observed in liver. We find that cooperative gene activation in combination with a TCF feedback can establish sharp borders of target gene expression in Wnt or APC gradients. In contrast, the iFFL renders gene expression independent of gradients of the upstream signaling components. Our subsequent analysis of carcinogenic pathway mutations reveals that their impact on gene expression is determined by the gene regulatory mechanism and the APC concentration of the cell in which the mutation occurs.

## Introduction

Wnt ligands are secreted signaling molecules that can generate concentration gradients across tissues (Zecca et al., 1996; Whangbo and Kenyon, 1999). In adult liver, time-independent gradients in Wnt signaling components, notably Wnt and APC (adenomatous polyposis coli), have been observed along the periportal-pericentral axis. In the periportal region of the mouse liver, high APC concentrations have been detected that decrease toward the pericentral region (Benhamouche et al., 2006; Torre et al., 2010). These changes in APC levels are paralleled by alterations in the expression profiles of Wnt/β-catenin target genes such as GS (glutamine synthetase), Lect2 (leukocyte cell-derived chemotaxin 2), or Gls2 (glutaminase 2) (Benhamouche et al., 2006; Braeuning et al., 2006). In addition to APC levels, it has been suggested that the available Wnt concentration is involved in the formation of the expression profiles (Benhamouche et al., 2006). The spatial differences in Wnt/β-catenin target gene expression have been suggested to contribute to the zonation of the liver, which may provide the basis for the liver’s ability to fulfill different metabolic functions (Jungermann and Kietzmann, 1996; Burke and Tosh, 2006; Colnot and Perret, 2011). Examples are urea formation, which is mostly observed in the periportal region, and glutamine synthesis, which occurs in the pericentral area (Jungermann and Kietzmann, 1996; Torre et al., 2010; Colnot and Perret, 2011).

The Wnt/β-catenin pathway exerts its control on gene expression mainly by regulating the concentration and the activity of β-catenin (Kimelman and Xu, 2006; Cadigan and Peifer, 2009; MacDonald et al., 2009). In resting cells, β-catenin is constantly produced and degraded via a destruction-complex-dependent mechanism. The destruction complex, which contains the scaffold proteins APC and Axin, mediates the phosphorylation and ubiquitination of β-catenin. A Wnt stimulus leads to the inhibition of the destruction complex. In consequence, less β-catenin is degraded and more protein is able to enter the nucleus. There it regulates the expression of specific target genes. To fulfill its function as a transcriptional regulator, β-catenin interacts with members of the TCF family of transcription factors which comprises T-cell factors (TCF7, TCF7L1, and TCF7L2) and lymphoid enhancer-binding factor (LEF1).

A number of Wnt/β-catenin target genes have been identified (a regularly updated list can be found at http://www.stanford.edu/group/nusselab/cgi-bin/wnt/). However, detailed regulatory mechanisms of their expression have been suggested for only a few. Experiments have shown that the expression of E-cadherin is directly inhibited by the β-catenin/TCF complex (Jamora et al., 2003). A subgroup of target genes, e.g. patterning genes, has been proposed to be regulated by an incoherent feedforward loop (iFFL) motif (Goentoro and Kirschner, 2009). Different members of the TCF family have been reported to be negatively or positively regulated by Wnt/β-catenin signaling thereby giving rise to different types of feedback loops (Roose et al., 1999; Hovanes et al., 2001; Saegusa et al., 2005; Vadlamudi et al., 2005; Driskell et al., 2007). In addition to these β-catenin/TCF-dependent regulatory mechanisms, the action of co-regulators as well as the occurrence of posttranslational modifications have been suggested as contributors to the regulation of Wnt/β-catenin target gene expression (Hecht and Kemler, 2000; Archbold et al., 2012). Some of these co-regulators may regulate the switch of TCF between its repressing and activating functions (Archbold et al., 2012).

An aberrant activity of the Wnt/β-catenin pathway is associated with various diseases, most notably with different types of cancer (Clevers, 2006; Klaus and Birchmeier, 2008). Many mutations of pathway components lead to a constitutive activation of the Wnt/β-catenin pathway by interfering with the destruction-complex-dependent degradation of β-catenin and thereby changing target gene expression. For instance, mutations in APC have been detected in 80% of colorectal cancer. Mutations in β-catenin occur with a frequency of 20–30% in hepatocellular carcinoma and up to 65% in hepatoblastomas. Hepatic cancer is also associated with mutations in Axin (Giles et al., 2003).

In recent years, several mathematical models of the Wnt/β-catenin pathway have been developed, reviewed in Kofahl and Wolf (2010). They range from detailed models which take into account the molecular interactions of the pathway’s components (Lee et al., 2003), to very condensed descriptions, which aim to reproduce the general dynamics of the signaling pathway (Mirams et al., 2010). These models have been used to investigate the interplay between the adhesive and transcriptional functions of β-catenin (van Leeuwen et al., 2007) as well as the effects of carcinogenic mutations of pathway components (Cho et al., 2006). A multitude of modeling approaches in different biological contexts has been focused on gradients in order to understand how gradients are created, maintained, and interpreted by cells (Gurdon and Bourillot, 2001; Jaeger et al., 2008; Meinhardt, 2009; Nahmad and Lander, 2011; Wolpert, 2011). However, the impact of gradients of Wnt growth factors or downstream pathway components on Wnt/β-catenin target gene expression has not yet been addressed by modeling approaches.

In our theoretical approach, we aim to understand the contribution of different gene regulatory mechanisms to mRNA profiles of Wnt/β-catenin target genes in the presence of time-independent Wnt or APC concentration gradients as observed in adult liver. To this end, we develop a minimal mathematical model for Wnt/β-catenin signal transduction and extend it by different gene regulatory mechanisms that have been proposed in the literature for Wnt/β-catenin target genes. In particular, (i) an activation mechanism, (ii) an inhibition mechanism, and (iii) an iFFL are investigated. Moreover, we consider the impact of an additional possible feedback via TCF. By simulating the effects of pathway mutations, we furthermore compare target gene mRNA concentrations under wild type and carcinogenic conditions. Our study demonstrates that the combination of specific gene regulatory mechanisms and a gradient of either Wnt or APC is sufficient to generate the distinct target gene expression patterns that have been observed in the liver.

## Results

### Model development

#### Minimal model of Wnt/β-catenin signal transduction and target gene expression

**Figure 1:**
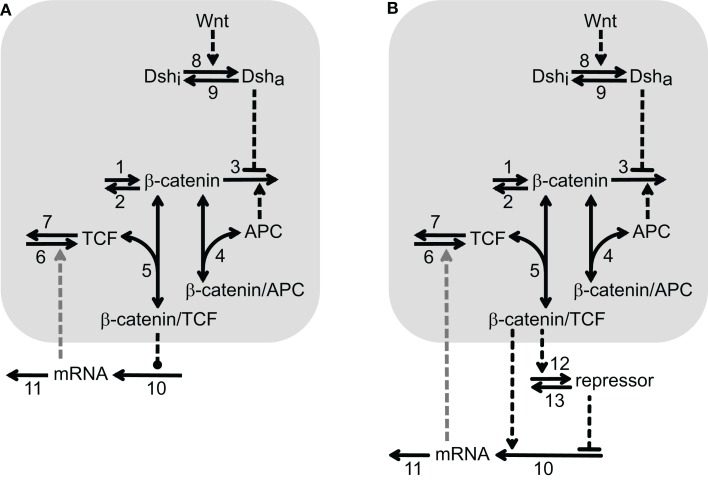
**Two reaction schemes of the minimal model illustrating different mechanisms of Wnt/β-catenin target gene regulation**. The signal transduction module is shared by **(A,B)** and highlighted by a light gray background. It consists of the reactions 1–9 that are explained in the text. The number next to the arrow denotes the particular reaction. One-headed arrows denote reactions taking place in the indicated direction. Double-headed arrows illustrate binding reactions. Dashed arrows represent activation steps and inverted, dashed “T”s denote inhibition. The gray, dashed arrow connecting the mRNA and reaction 6 indicates the possible feedback considered in Section “Transcriptional feedback regulation via TCF family members”. The two schemes differ in their downstream target gene regulation motif. **(A)** Direct regulatory mechanisms: the dashed line ending with a dot between β-catenin/TCF complex and reaction 10 indicates that the β-catenin/TCF complex regulates the mRNA production either by a direct activating or repressing mechanism (see Sections “Activation of Wnt/β-catenin target genes” and “Repression of Wnt/β-catenin target genes”, respectively). **(B)** Incoherent feedforward loop: β-catenin/TCF complexes induce the expression of a repressor of the mRNA production, which counteracts the simultaneous direct activation of mRNA expression by β-catenin/TCF complexes in reaction 10, see Section “Wnt/β-catenin target gene regulation by an incoherent feedforward loop motif”.

Various aspects of the Wnt/β-catenin pathway have been investigated in theoretical studies (Kofahl and Wolf, 2010). However, the available models either do not focus on possible regulation of downstream target gene expression and/or miss pathway components which are of importance in our analysis. We therefore develop a minimal model for the Wnt/β-catenin signaling pathway that enables us to easily combine signal transduction with different mechanisms of downstream target gene regulation. We consider individual target genes because gene regulatory networks related to Wnt/β-catenin signaling have not yet been described in the liver. Since we focus on transcriptional rather than translational regulation, only the mRNA of the target genes is included in the minimal model. Two schemes of the minimal model with identical Wnt/β-catenin signal transduction modules, but different downstream gene regulatory motifs, are shown in Figures 1A,B.

The Wnt/β-catenin signal transduction module of our minimal model consists of β-catenin, TCF, APC, the β-catenin/TCF complex, the β-catenin/APC complex as well as active and inactive Dishevelled (Dsh_a_ and Dsh_i_, respectively). β-Catenin is constantly produced (reaction 1) and degraded in an APC-independent as well as an APC-dependent manner (reactions 2 and 3, respectively). β-Catenin can reversibly form complexes with APC (reaction 4) and TCF (reaction 5). TCF is produced and degraded (reactions 6 and 7, respectively) while the total concentration of APC is assumed to remain constant within the considered time period. The APC-dependent degradation of β-catenin is inhibited by activated Dishevelled. Dishevelled is activated by a Wnt stimulus (reaction 8) and can become deactivated again (reaction 9). Like APC, Dsh obeys a conservation relation.

The gene regulatory mechanisms investigated here can be grouped into two different representations illustrated in the two model schemes (Figures 1A,B). In Figure 1A, the β-catenin/TCF complex directly regulates the transcription of target gene mRNA (reaction 10). Both direct positive and direct negative regulatory mechanisms are considered in this study. In Figure 1B, the motif of the iFFL is coupled to the signal transduction module. In comparison to the scheme in Figure 1A, an additional repressor is involved in this mechanism. Its synthesis is induced by the β-catenin/TCF complex (reaction 12) and it is independently degraded via reaction 13. The repressor inhibits the production of target gene mRNA, thereby counteracting direct mRNA activation by the β-catenin/TCF complex (reaction 10). The mRNA is degraded in all cases via reaction 11 (Figures 1A,B). In Section “Transcriptional feedback regulation via TCF family members”, we investigate the implications of an additional feedback acting in combination with the regulatory mechanisms. This feedback is indicated by a gray dashed arrow from the mRNA to the TCF production (reaction 6) in Figures 1A,B.

Overall, the signal transduction module consists of five ordinary differential equations (ODEs) and two conservation relations (see Section “Model equations and parameters” in the Appendix, Eqs A1–A16). In the model presented in Figure 1A, one additional ODE is included to account for the mRNA dynamics. In the model given in Figure 1B, two additional ODEs, one accounting for the repressor dynamics and one describing the mRNA dynamics, are coupled to the signal transduction module. The kinetic parameters of the signal transduction module are set in such a way that a strong accordance between the minimal and the quantitative model by Lee et al. (2003) (hereafter referred to as the detailed model) regarding the β-catenin steady state and response to a constant Wnt stimulus is achieved, see Section “Comparison of the minimal and the detailed model”. Most of the kinetic parameters as well as the total concentrations of APC and Dsh (APC^tot^ and Dsh^tot^, respectively) are adopted from the detailed model. They are given in Tables A1 and A2 of Section “Model equations and parameters” in the Appendix and are used in all simulations. The expression of target gene mRNA downstream of the signaling module is described in a qualitative way since quantitative data are lacking so far. The steady state concentration of the mRNA is therefore set to an arbitrary value of about 0.68 nM under the condition of a total APC concentration of 100 nM and the absence of Wnt. This absolute mRNA concentration can easily be rescaled to more appropriate values once specific experimental data become available.

The analysis focuses on the consequences of gene regulatory mechanisms within a Wnt or APC gradient. To cover the conditions suggested to occur in the liver, the theoretical analyses are performed for a time-independent gradient of the Wnt and the total APC concentration. Quantitative data on the range and the spatial distribution of the concentration in these gradients are missing. To include all possible conditions, we vary the Wnt and APC concentrations over a wide range.

#### Comparison of the minimal and the detailed model

**Figure 2:**
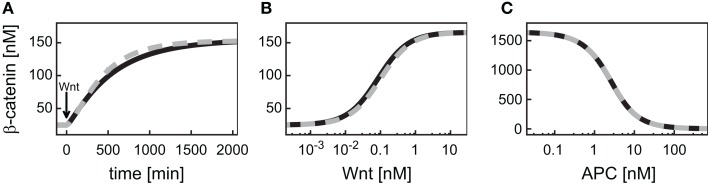
**Comparison of the dynamical properties of β-catenin in the minimal and the detailed model**. **(A)** Time courses of β-catenin in response to a constant Wnt stimulation. **(B,C)** Steady states of β-catenin for different levels of Wnt or APC, respectively, within corresponding concentration gradients. The simulations of the minimal (black, solid line) and the detailed model (gray, dashed line) are compared.

First, we compare the dynamics of β-catenin between the minimal model (black, solid line) and the detailed model (gray, dashed line; Figure 2). The unstimulated and the Wnt-stimulated steady state levels of β-catenin are almost identical in the minimal and the detailed model; the deviation is less than 1.5% in both cases (Figure 2A). The minimal model, moreover, reproduces the dependence of the β-catenin steady state concentration on the Wnt or total APC concentration (Figures 2B,C). In general, it holds that the β-catenin steady state concentration is higher the higher the Wnt concentration or the lower the APC concentration. Note that the β-catenin concentration range is considerably broader in the APC gradient than in the Wnt gradient (around 4–1646 nM compared to 25–166 nM β-catenin, respectively), as both components act differently on the degradation of β-catenin (reaction 3). We also observe a strong agreement between the two models in their β-catenin time courses upon pathway activation by a constant Wnt stimulus. In both models, the constant Wnt stimulus is realized by setting *Wnt* from 0 to 1 nM at time point *t* = 0 min. The β-catenin dynamics in response to a transient Wnt stimulus cannot completely be captured (not shown). Taken together, the minimal model very well reproduces the dynamical properties of β-catenin that are relevant for our analysis.

**Figure 3:**
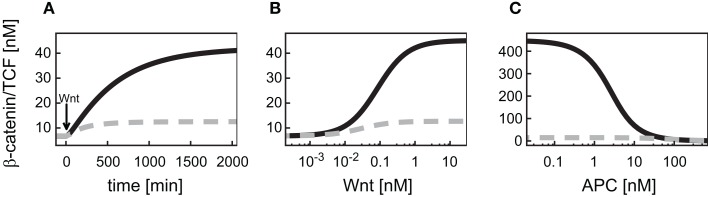
**Comparison of the dynamical properties of the β-catenin/TCF complex in the minimal and the detailed model**. **(A)** Time courses of the β-catenin/TCF complex in response to constant Wnt stimulation, and **(B,C)** steady states of the β-catenin/TCF complex in gradients of Wnt and APC concentration, respectively, are compared between the minimal (black, solid line) and the detailed model (gray, dashed line).

In the detailed model, the TCF concentration obeys a conservation relation. Since here we also address a possible feedback mechanism via the transcriptional regulation of TCF, a turnover of TCF is included in our signaling module. This modification has implications for the steady states and dynamics of the β-catenin/TCF complex. While the unstimulated steady state of the β-catenin/TCF complex differs less than 1% between the minimal and the detailed model, the complex concentration increases more strongly upon Wnt stimulation in the minimal model, resulting in a higher stimulated steady state (Figure 3A). In both models the steady states of the β-catenin/TCF complex increase with an increasing Wnt or a decreasing APC concentration (Figures 3B,C). Overall, due to the restriction of the total TCF concentration in the detailed model, the concentration range of the β-catenin/TCF complex is much smaller compared to the minimal model.

Wnt and APC are differentially integrated by the signaling module. This has consequences for the signaling readout. First, the β-catenin as well as the β-catenin/TCF complex steady state concentration changes in opposite directions along the Wnt and the APC gradient. Secondly, the signal transduction module restricts the effective concentration ranges of the gradients differently, outside of which a further increase or decrease of the concentration of Wnt or APC does not lead to a significant change of the β-catenin concentration (see Figures 2B,C). A change in APC concentration can directly modulate β-catenin degradation (reaction 3; Eq. A10). Therefore, the β-catenin steady state is only limited by the ratio of its production to its alternative degradation rate constants (Eqs A8 and A9, respectively). In contrast, a change in the Wnt concentration is not linearly passed to the β-catenin degradation (reaction 3), but has an indirect effect via the activation of Dsh. Dsh obeys a conservation relation (Eq. A6) which leads to a limitation of the Wnt concentration range that influences the β-catenin degradation.

### Analysis of target gene expression

#### Wnt/β-catenin target gene regulation by an incoherent feedforward loop motif

**Figure A1:**
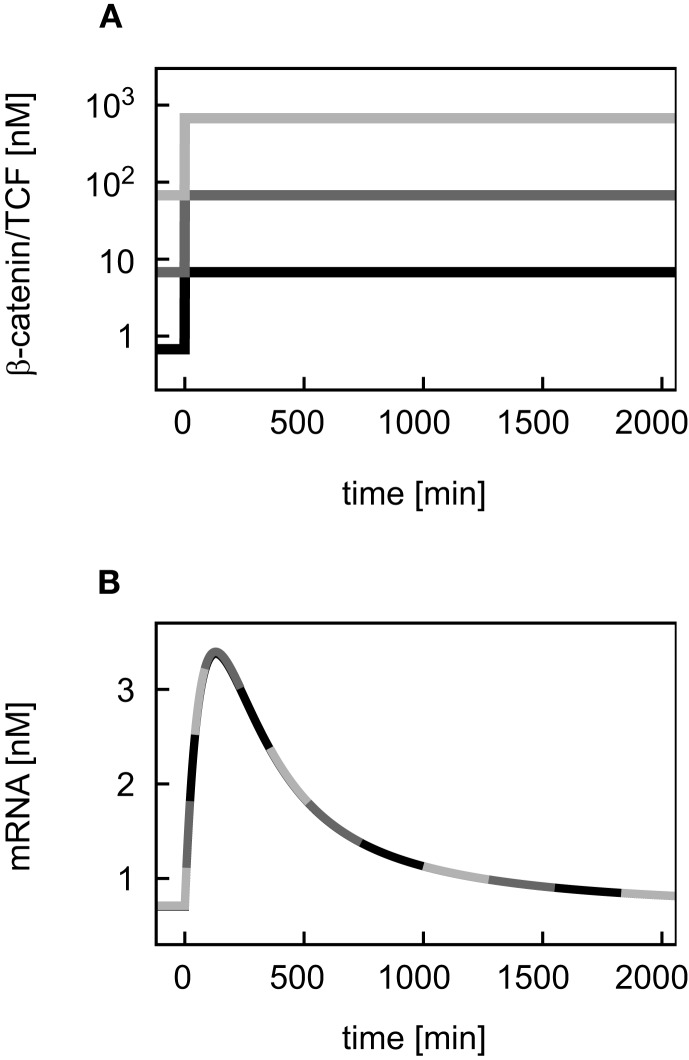
**Input (β-catenin/TCF) and output (mRNA) time courses of the iFFL motif**. **(A)** A stepwise increase of the β-catenin/TCF concentration leads to **(B)** a perfect adaptation of the target gene mRNA. The dynamics of the mRNA is independent of the initial absolute concentration of β-catenin/TCF if the same fold change in the β-catenin/TCF level is induced.

It has been proposed that a subgroup of Wnt target genes, in particular patterning genes, is regulated by an iFFL motif (Goentoro and Kirschner, 2009; Goentoro et al., 2009). We investigate the impact of this motif using our model (Figure 1B). We chose the parameters of the iFFL motif in a way that the motif reproduces all the properties described in Goentoro et al. (2009); parameters are given in Table A3 of Section “Model equations and parameters” in the Appendix. These are the ability to generate a perfect adaptation of the mRNA concentration and the independence of the mRNA response to the initial absolute concentration of β-catenin/TCF (Figure A1 in the Appendix). It has been shown that the iFFL motif meets these two requirements only for parameters chosen from a certain parameter range (Goentoro et al., 2009).

**Figure 4:**
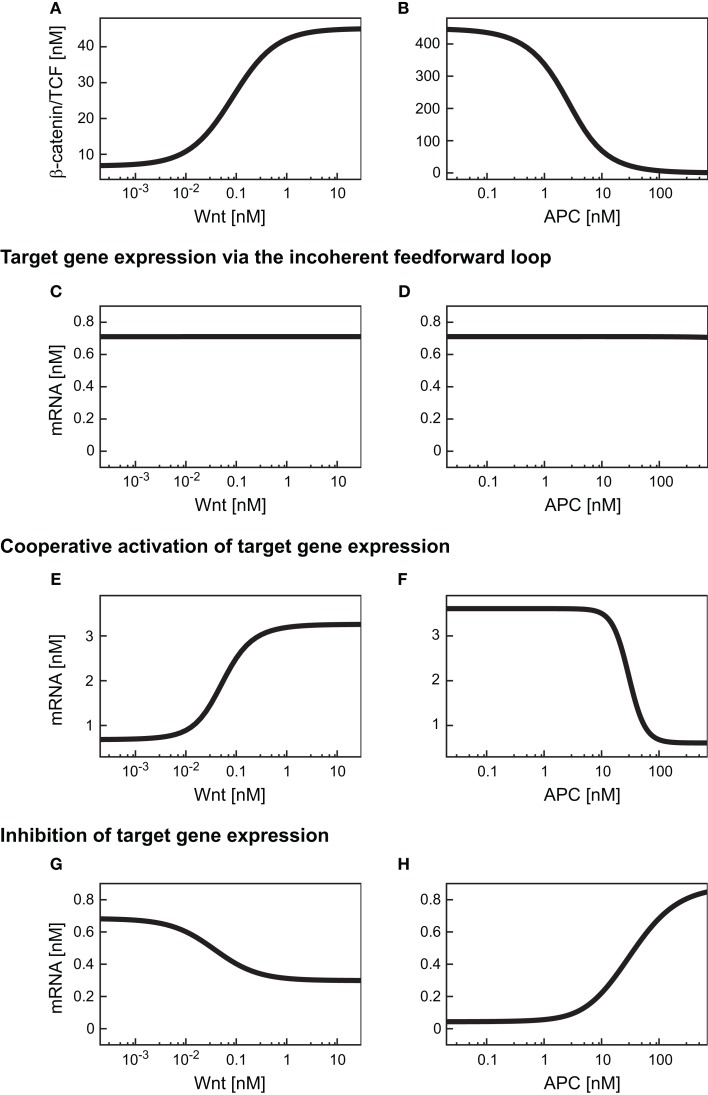
**Expression levels of differentially regulated target genes in a Wnt or an APC concentration gradient**. **(A,B)** β-Catenin/TCF steady states within the Wnt or the APC concentration gradient. **(C–H)** Steady state concentrations of target genes induced by an iFFL motif **(C,D)**, induced by cooperative activation with the Hill coefficient *n* = 3 **(E,F)**, and upon inhibition **(G,H)** are shown within **(C,E,G)** a Wnt gradient or **(D,F,H)** an APC gradient.

Having verified that the iFFL in the minimal model reproduces these characteristics, we next analyzed the consequences of this motif on the mRNA steady state within a Wnt or an APC gradient. Our simulations show that an increase in the Wnt concentration along a gradient results in an increase of the β-catenin/TCF level (Figure 4A). In contrast to the response of β-catenin/TCF, the mRNA steady state concentration remains almost constant along the Wnt gradient (Figure 4C). Similar to our observations with the Wnt gradient, levels of β-catenin/TCF change along the APC concentration gradient (Figure 4B). Again, the iFFL motif renders the mRNA steady state concentration robust over a wide APC concentration range (Figure 4D). Overall, the iFFL motif allows for a constant target gene expression if the parameters are chosen in the way described above.

#### Activation of Wnt/β-catenin target genes

**Figure 5:**
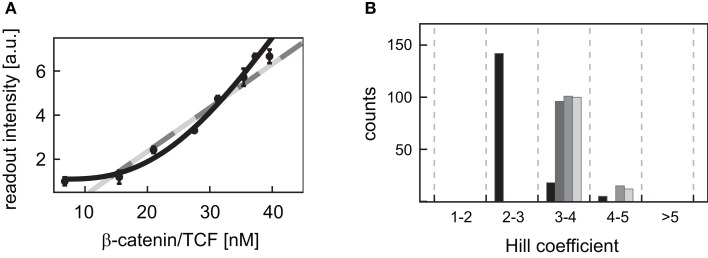
**Fitting of different kinetic expressions to reporter construct data**. **(A)** The readout intensity data of the reporter construct with three TBEs is plotted with standard deviations; data provided by Biechele and Moon (2008). The respective linear activation function (light gray), Michaelis–Menten activation function (dark gray), and Hill-type activation function (black) realizing the best fit (i.e., yielding the minimal log-likelihood function value) are shown. The fits of the linear and the Michaelis–Menten activation lie on top of each other. **(B)** The distributions of the fitted Hill coefficients are shown for the four reporter constructs. Only those Hill coefficients of fits are taken into account, whose log-likelihood function value deviates at most 20% from the log-likelihood function value of the best fit. The constructs with 3, 8, transiently transfected 12 TBEs, and stable integrated 12 TBEs are color-coded in black, dark gray, gray, and light gray bars, respectively.

**Figure A2:**
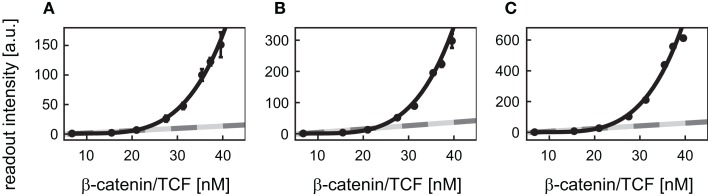
**Comparison of the best fit functions**. The readout intensity of the reporter constructs with 8, 12 (transfected), and 12 (integrated) TBEs **(A–C)** are plotted with standard deviations. The linear activation function (light gray), the Michaelis–Menten activation function (dark gray), and the Hill-type activation function (black) realizing the best fit (i.e., yielding the minimal log-likelihood function values) are shown for each reporter construct. Note, that the fits of the linear and the Michaelis–Menten activation lie on top of each other. The experimental data was kindly provided by Biechele and Moon (2008).

For many Wnt target genes, an induction of their expression upon activation of the Wnt/β-catenin pathway has been reported. Examples are Axin-2 as well as the liver-specific Wnt target genes OAT (ornithine aminotransferase) and Glt1 (glutamate transporter 1) (Cadoret et al., 2002; Jho et al., 2002). In the promoters of many Wnt target genes, multiple TCF/LEF binding elements (TBEs) have been detected. For instance, eight TBEs have been identified in the promoter and first intron of Axin-2 (Jho et al., 2002), and three TBEs have been reported for the liver-specific Wnt target gene Lect2 (Ovejero et al., 2004). Multiple TBEs open the possibility of cooperative target gene activation. However, detailed information on the activation of target gene expression is limited. To gain insights into possible activation mechanisms, we analyzed published experimental data of β-catenin reporter constructs (Biechele and Moon, 2008). In these experiments, the responses to different Wnt concentrations of reporter constructs with 3, 8, and 12 TBEs have been compared. In our approach, we considered three possible kinetics for the transcription rate: (i) linear mass action kinetics, (ii) Michaelis–Menten kinetics, and (iii) Hill-type activation. We fitted the kinetics (i), (ii), and (iii) to the data and compared the calculated values of the corrected Akaike information criterion (AICc; see Section “Fitting of reporter data” in the Appendix) (Hurvich and Tsai, 1995; Lukacs et al., 2010). The analysis revealed that the data for all four constructs are best explained by Hill-type functions (Figure 5A; Figure A2 in the Appendix), with Hill coefficients *n* higher than 1 (Figure 5B) suggesting a cooperative regulation of these reporters. We furthermore observed that the medians of the Hill coefficients for the construct with 8 TBEs and those with 12 do not vary significantly (3.9, 4.0, and 3.9, respectively) although the number of TBEs increases by 50%. The median of the construct with three TBEs is smaller than the other three values (2.6). This analysis suggests that the expression of the β-catenin reporter constructs occurs in a cooperative manner.

We next investigated the consequences of cooperative activation on target gene mRNA expression within a Wnt or an APC concentration gradient. To this end, we used the overall minimal model including signal transduction and gene expression, with the mRNA production rate (*v*_10_) given by the Eq. A25 (Section “Model equations and parameters” in the Appendix). A Hill coefficient of *n* = 3 as derived from the analysis of the reporter data is used (see Figure 5B and Section “Fitting of reporter data” in the Appendix). All kinetic parameters are given in Table A4 of Section “Model equations and parameters” in the Appendix. Our simulations show that the target gene mRNA concentration increases with increasing Wnt or decreasing APC concentrations (Figures 4E,F). There exist distinct ranges of Wnt and APC concentrations in which the mRNA levels strongly change. Outside these ranges, changes in Wnt or APC levels do not significantly affect mRNA levels. Furthermore, we observe that the APC gradient creates a slightly larger mRNA concentration range than the Wnt gradient.

**Figure 6:**
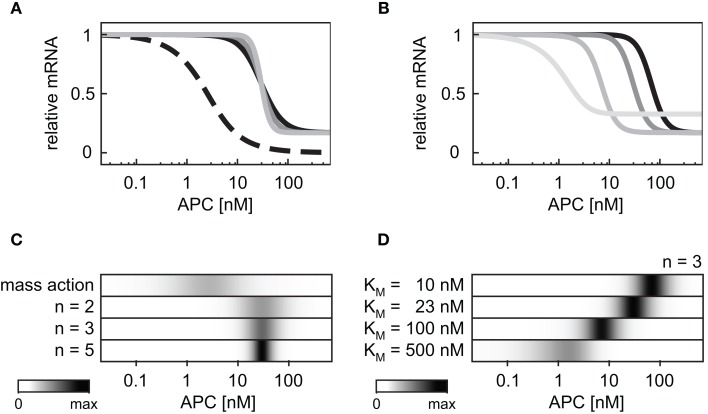
**Impact of the mRNA expression kinetics and their parameters on the mRNA concentration profile within an APC concentration gradient**. **(A)** The steady state concentration of the target gene mRNA is compared assuming a mass action activation (dashed black line) and cooperative activation with Hill coefficients *n* = 2, 3, and 5 (black, dark gray, and light gray, respectively) and *K*_M_ = 23 nM in all three cases. For each curve the steady state is normalized to the respective maximal mRNA concentration. **(B)** The steady state concentration of the target gene mRNA is compared assuming cooperative activation with *K*_M_ values of 10, 23, 100, and 500 nM (black, dark gray, gray, and light gray, respectively). The Hill coefficient is set to *n* = 3 in all cases. For each curve the steady state is normalized to the respective maximal mRNA concentration. **(C,D)** The absolute value of the concentration changes along the curves of **(A,B)**, respectively, are compared. The maximal calculated change in each panel is color-coded in black.

Next we compared the effects of the cooperative target gene activation with Hill coefficients *n* = 2, 3, or 5 to that of a linear transcription rate in the APC gradient. The linear mRNA production rate (*v*_10_) is described by Eq. A24 (Section “Model equations and parameters” in the Appendix). We observed that the possible range of the relative mRNA concentration within the APC gradient is narrower for the cooperative activation than for a linear activation (Figure 6A) due to the saturation of the Hill-type activation. Figure 6C shows that the APC concentration range in which the mRNA levels change is broad in the case of the mass action kinetics (Figure 6C, first line) whereas the range is narrow in the case of cooperative activation (Figure 6C, lines 2–4). The larger the Hill coefficient is, the smaller the APC concentration range in which the mRNA levels change. The absolute APC concentration for which the mRNA levels change decreases the larger the *K*_M_ value is (Eq. A25), as shown in Figures 6B,D. If the *K*_M_ value becomes larger than the maximal possible β-catenin/TCF complex concentration in our model (*K*_M_ > 450 nM), *K*_M_ additionally influences the width of the APC concentration range in which the mRNA levels change. Under these conditions, *K*_M_ also reduces the possible relative mRNA concentration range.

#### Repression of Wnt/β-catenin target genes

E-cadherin is a well-known example of negatively regulated Wnt target genes (Huber et al., 1996; Jamora et al., 2003). Also several liver-specific Wnt target genes such as Gls2, Arg1 (arginase 1), and Cps1 (carbamoyl-phosphatase 1) have been shown to be repressed by Wnt/β-catenin signaling (Benhamouche et al., 2006). In most cases the mechanisms mediating the repression have not been characterized in detail. In the case of E-cadherin, a direct repression has been demonstrated (Huber et al., 1996; Jamora et al., 2003). To investigate a direct negative regulation mechanism, we integrated an inhibitory function in the rate of transcription (Eq. A28; the kinetic parameters are given in Table A5 of Section “Model equations and parameters” in the Appendix).

First, the consequences of a negative regulation of a target gene within the minimal model were analyzed within a Wnt gradient (Figure 4G). We observed a decreasing steady state concentration of the mRNA along the increasing Wnt gradient. However, the mRNA level is kept in a very restricted concentration range. The underlying reason is the limitation of the inhibition by the available β-catenin/TCF concentration in the gradient (Figure 4A).

**Figure 7:**
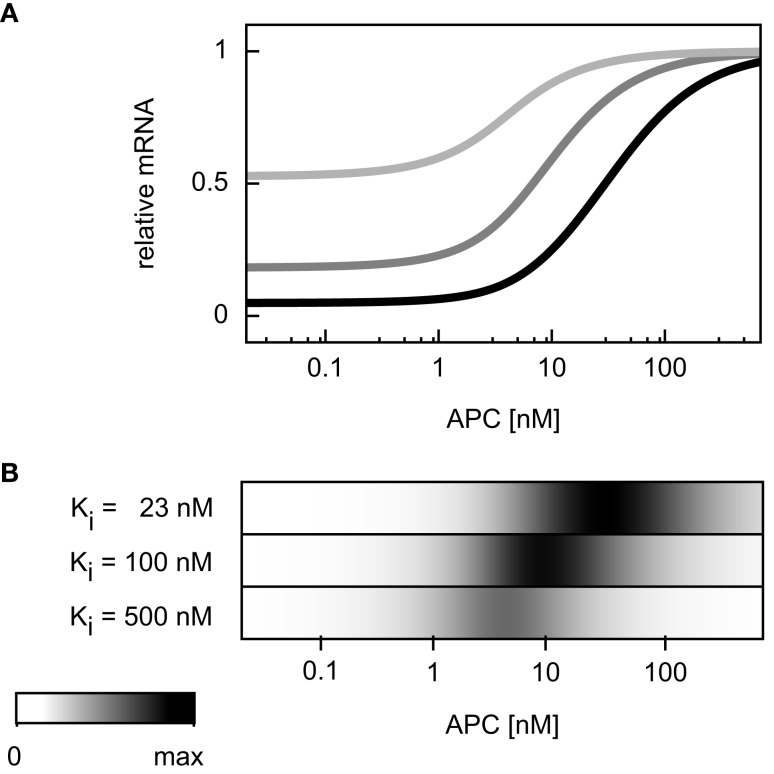
**Dependence of the critical APC concentration for target gene induction on the inhibition constant**. **(A)** The steady state concentration of target gene mRNA is compared assuming different values of the inhibition constant *K*_i_: 23, 100, and 500 nM (black, dark gray, and light gray, respectively). For each curve, the steady state is normalized to the respective maximal mRNA concentration. **(B)** The absolute values of the concentration changes along the curves of **(A)** are plotted. The maximal calculated change is color-coded in black.

In the APC gradient a critical concentration threshold of APC must be reached to induce a target gene response (Figure 4H). At APC concentrations above this threshold, the mRNA levels increase with the APC concentration until saturation is reached at very high APC levels. The range of the mRNA concentration covered by the APC gradient is larger than that by the Wnt gradient (Figures 4H,G). The inhibition constant *K*_i_ (Eq. A28) strongly determines the critical APC concentration above which the mRNA level react to changes in the APC concentration (Figure 7). The larger *K*_i_ is, the smaller the critical concentration threshold. Furthermore, *K*_i_ influences the possible relative mRNA concentration range; larger *K*_i_ values reduce this range.

#### Transcriptional feedback regulation via TCF family members

The Wnt/β-catenin signaling pathway is regulated by several types of transcriptional feedback (Logan and Nusse, 2004). Cases of negative feedback via Axin-2 or Dickkopf (DKK) are prominent examples that have been investigated both experimentally (Leung et al., 2002; Lustig et al., 2002; Niida et al., 2004) and theoretically (Wawra et al., 2007; Goldbeter and Pourquie, 2008; Jensen et al., 2010). Here, we concentrate on possible transcriptional feedbacks via TCF family members (Roose et al., 1999; Hovanes et al., 2001; Saegusa et al., 2005; Vadlamudi et al., 2005; Driskell et al., 2007). To analyze the impact of this transcriptional feedback on Wnt/β-catenin target gene expression, we replaced the constant TCF production rate (Eq. A13) with an mRNA-dependent translation rate (Eq. A30; parameter given in Table A6 of Section “Model equations and parameters” in the Appendix). In the following we study the effect of the different target gene expression mechanisms in the presence of a TCF feedback, beginning with the iFFL motif.

**Figure 8:**
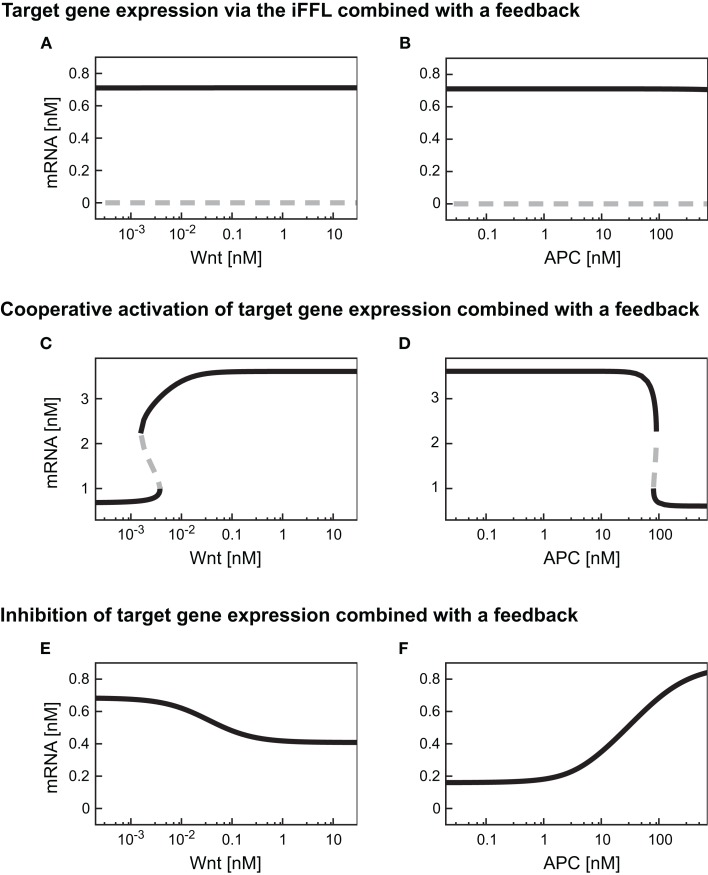
**Steady state responses of differently regulated target genes in the presence of a TCF feedback**. Steady state responses of target genes regulated by an iFFL motif **(A,B)**, in a cooperative manner with the Hill coefficient *n* = 3 **(C,D)**, and by inhibition **(E,F)** are shown within **(A,C,E)** a Wnt gradient or **(B,D,F)** an APC gradient. The color-code denotes stable steady states as black solid lines and instable steady states as gray dashed lines.

As described in Section “Wnt/β-catenin target gene regulation by an incoherent feedforward loop motif” the iFFL motif renders the target gene expression level independent of the Wnt concentration present. The addition of a feedback (i.e., replacing Eq. A13 by Eq. A30) does not change this observation (Figure 8A). The comparison of Figures 4C and 8A shows that the target gene steady state levels are not changed by the addition of the feedback. These observations also hold for the mRNA level within the APC gradient (Figure 8B). The feedback leads to the occurrence of a second steady state solution at very low mRNA concentrations in both gradients. However, this steady state is instable and can therefore not be detected in experiments.

By combining the cooperative activation of mRNA production with the feedback mechanism, a discontinuous dependence of mRNA expression level on the Wnt or APC concentration can arise (Figures 8C,D). At low Wnt concentrations, mRNA levels remain low until a critical Wnt concentration is reached (Figure 8C). If the Wnt concentration exceeds this critical value, the mRNA concentration rises instantaneously to a high level. Beyond the critical Wnt concentration, a further increase of the Wnt concentration only leads to a minor increase of the mRNA expression level. A decrease of the Wnt concentration results in the drop of the mRNA concentration to the initial level. This drop also occurs instantaneously, but at a lower Wnt concentration than the critical concentration mentioned above. In the range between the two critical Wnt concentrations, cells of low and high mRNA expression levels may coexist. Such a bistability, that is, the coexistence of low and high mRNA-expressing cells, is also possible in an APC gradient (Figure 8D).

The comparison of the absolute mRNA concentrations in the models with and without the feedback (Figures 8C and 4E, respectively) reveals that within the Wnt gradient, the mRNA can reach higher maximal levels in the presence of the feedback compared to its absence. In the APC gradient, the mRNA concentration range does not significantly change due to the feedback (Figures 4F and 8D). The reason is that for low APC levels, the β-catenin/TCF concentration is already much larger than the *K*_M_ value in the transcription rate (Eq. A25). A further increase of the β-catenin/TCF concentration due to the feedback does therefore hardly affect the transcription rate and consequently the mRNA concentration.

**Figure 9:**
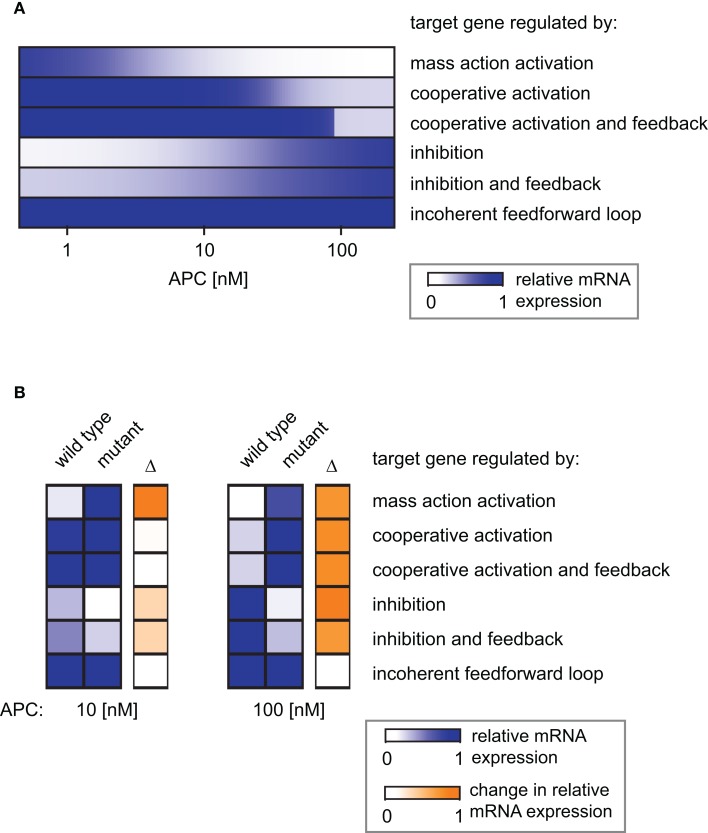
**Simulation of the distinct target gene expression patterns within an APC gradient**. **(A)** The relative expression profiles along an APC gradient are simulated for different target genes each subject to a different regulatory mechanism as indicated. **(B)** Relative mRNA expression at two positions in the APC gradient (*APC* = 10 nM, left, and *APC* = 100 nM, right) for the six indicated gene regulatory mechanisms. The relative expression under wild type and mutated conditions is shown as well as the absolute difference (Δ) as a measure of the impact strength of the mutation (color-coded in blue and orange, respectively). The mutation exclusively affects the APC-dependent β-catenin degradation (reaction 3); the parameter *k*_3_ in Eq. A10 is set to 1% of its original value in the mutant case.

The combination of transcriptional target gene inhibition with a feedback mechanism creates a negative feedback loop. Under certain conditions, negative feedback loops are able to create limit cycle oscillations around an instable steady state. However, we detected only stable steady state solutions in our minimal model for the given kinetic parameters (Figures 8E,F). By comparing the model with and without the feedback we observe that the feedback reduces the concentration range of the target gene mRNA. In particular, at high Wnt concentrations, the mRNA concentration is higher in the presence of the feedback than in its absence (Figures 8E and 4G, respectively). This implies that the feedback mechanism overall weakens the inhibitory effect of β-catenin/TCF on the mRNA expression. A similar effect can be observed in the APC gradient. At low APC concentrations the presence of the feedback weakens the repressive effect of the β-catenin/TCF complex on the mRNA expression. This results in a higher mRNA concentration in the presence compared to the absence of the feedback mechanism (Figures 8F and 4H). Despite its impact on the mRNA concentration, the feedback mechanism hardly alters the concentration range along the APC gradient in which the mRNA concentration is sensitive to APC concentration changes (Figure 9A, lines 4 and 5).

#### The effects of carcinogenic mutations on target gene expression

Mutations in Axin or β-catenin frequently lead to a constitutive activation of Wnt/β-catenin signaling in hepatic cancer cells (Giles et al., 2003; Takigawa and Brown, 2008). These mutations often interfere with the APC-dependent degradation of β-catenin. In our model, they all affect reaction 3 and can be simulated by changing the rate constant of that reaction. To realize one representative mutation, we set the rate constant to 1% of its original value. In Figure 9B, the mRNA expression level of the mutant situation is simulated at two positions in the APC gradient and compared to the corresponding wild type situation.

For an APC concentration of 100 nM, the mRNA levels of all but the iFFL-regulated genes are strongly changed by the mutation. In contrast, with an APC concentration of 10 nM, the same mutation strongly affects only mass-action-regulated target genes. Expression levels of negatively regulated target genes are weakly changed by the mutation. The mutation does not significantly augment the mRNA levels of cooperatively activated target genes, since the critical APC concentration for target gene activation has been passed, resulting in already high mRNA expression. We observe that at 100 nM as well as 10 nM APC, iFFL-regulated gene expression is not influenced by the mutation. The results show that the impact of a mutation on the mRNA concentration depends on both the APC content of the cell and the specific regulatory mechanism of the target gene.

## Discussion

In this study, we qualitatively analyzed the implications of different gene regulatory mechanisms in time-independent gradients of concentrations of either Wnt or APC. To this end, we developed a minimal model of Wnt/β-catenin signal transduction and extended it to different regulatory mechanisms of target gene expression. Our analysis demonstrated that combining these mechanisms with a gradient in the concentration of either Wnt or APC is sufficient to generate spatially distinct target gene expression patterns as have been experimentally observed in liver (Benhamouche et al., 2006; Braeuning et al., 2006). The gene regulatory mechanism and the available concentration of APC also determine the impact of a mutation on the target gene expression.

The development of an integrative model including signal transduction and different gene regulatory mechanisms has allowed us to investigate how the concentration of Wnt or APC is translated via the signaling processes and transcriptional regulation into target gene expression. So far, most studies have focused either on the impact of a gradient in the context of the signal transduction (Melen et al., 2005; Murray et al., 2010) or in the context of gene regulatory networks (Balaskas et al., 2012) but did not interlace both aspects. Our analysis showed that the differential integration of the Wnt and the APC concentration into the signaling processes has two consequences. First, the signaling pathway transmits the Wnt and APC gradient into opposing β-catenin/TCF complex concentration profiles (Figures 3B,C). Second, the signaling processes limit the possible concentration range of the transcriptional active β-catenin/TCF complex along the gradients. By this means, the possible Wnt or APC concentration range is confined to a narrower effective range that influences target gene mRNA levels.

Applying our model, we investigated how the different gene regulatory mechanisms influence target gene expression within Wnt or APC concentration gradients. Our simulations showed that direct activation and inhibition mechanisms for target gene mRNA can generate opposite expression patterns (Figure 9A, lines 1 and 4, respectively, for an APC gradient). Such opposing expression profiles have also been detected by *in situ* hybridization experiments of liver sections. In regions with high expression of positively regulated target genes such as Lect2 and Axin-2, the expression of negatively regulated target genes such as Gls2 and Arg1 was low and *vice versa* (Colnot and Perret, 2011).

Until now, details on the molecular regulation of target gene expression are not available in the literature. For the characterization of the activation of gene expression, we investigated different possible activation kinetics using reporter construct data (Biechele and Moon, 2008). Our analysis indicated that the expression of these constructs occurs in a cooperative manner; the data were better approximated by Hill-type kinetics than by linear or Michaelis–Menten kinetics (Figure 5A; Figure A2 and Table A7 in Appendix). The Hill coefficients, which are a measure of cooperativity, were quantified to be in the range of 2–5. Interestingly, the Hill coefficients do not depend linearly on the number of TBEs in the constructs. While an increase in the number of TBEs from 3 to 8 is accompanied by a considerable increase in the Hill coefficient, an increase from 8 to 12 TBEs hardly changes the Hill coefficient. One might speculate that a critical number of TBEs is necessary to realize maximal cooperativity; any additional TBE may affect the readout intensity but not cooperativity. Whether these results also hold for natural Wnt/β-catenin target genes has to be verified experimentally. An indication for the cooperative activation of natural Wnt/β-catenin target genes is provided by site-directed mutation studies in the siamois promoter that harbors three TBEs. The elimination of one TBE weakly reduced the response of target gene expression to stimulation while the elimination of any pair of sites reduced most of the response (Brannon et al., 1997).

Taking cooperative activation of target gene expression into account, the simulations revealed that the Hill coefficient correlates with the steepness of the change in the mRNA level (Figures 6A,C). Furthermore, we demonstrated that a combination of a cooperative activation mechanism with *n* = 3 and a feedback via TCF is sufficient to create an all-or-none response for target gene expression (Figure 9A, line 3). Thus, cooperative activation with large Hill coefficients, or in combination with feedback, could create sharp borders in the zonation of target gene expression. Positive feedbacks and cooperativity have already been described to establish switch-like responses in other systems (Tyson et al., 2003; Melen et al., 2005). Whether the sharp expression profile of liver-specific Wnt target genes such as GS (Gebhardt et al., 2007; Colnot and Perret, 2011) is established by a cooperative regulation and to what extent additional mechanisms contribute remains to be investigated. Generally, several additional mechanisms are discussed in the literature as contributors to the zonation of target gene expression in the liver, e.g., (i) the integration of different signaling pathways (Hailfinger et al., 2006; Gebhardt and Hovhannisyan, 2010; Archbold et al., 2012), (ii) varying microenvironments (Jungermann and Kietzmann, 1996; Torre et al., 2010; Colnot and Perret, 2011), and (iii) differences in the activation of the Wnt/β-catenin pathway (Torre et al., 2010; Colnot and Perret, 2011). In the case of GS, a contribution of other signaling pathways induced by oxidative stress or growth hormones has been suggested (Gebhardt and Hovhannisyan, 2010).

Different target genes regulated by the same mechanism may still be expressed in different regions along the periportal-pericentral axis in the liver. Examples are the two negatively regulated target genes Gls2 and Arg1. While Gls2 is only expressed in the proximal periportal region, Arg1 is expressed in the proximal and distal periportal area (Benhamouche et al., 2006). Our theoretical analysis showed that the critical Wnt or APC concentration, at which changes in mRNA levels occur, strongly depends on the parameters *K*_i_ in target gene repression or *K*_M_ in target gene activation. Different parameter values shift the APC concentration range in which changes of gene expression occurs (Figures 6B,D as well as Figure 7). Thus, differences in kinetic parameters might be an explanation for the different expression areas of Gls2 and Arg1.

While the activation and inhibition mechanisms discussed above cause changes in the mRNA level along a Wnt or an APC gradient, the iFFL motif renders the steady state of the target gene independent of the gradient (Figure 9A, line 6). The steady state concentration of the target gene throughout the gradient is also insensitive to the addition of the feedback via TCF (Figures 8A,B). Provided that the iFFL motif exists and operates in hepatic gene regulation, it might be a mechanism to neutralize the impact of the gradients in order to establish constant expression levels of genes involved in zonation-independent processes. In that respect, it is interesting to note that not all metabolic processes are locally confined in the liver; e.g. the synthesis of serum proteins (Colnot and Perret, 2011).

Signal transduction in the Wnt/β-catenin pathway is perturbed in several types of cancer (Giles et al., 2003). We analyzed mutations in Axin and β-catenin that are frequently detected in hepatic cancer. Our study demonstrated that the impact of the mutation on the mRNA concentration depends on the specific gene regulatory mechanism as well as the APC concentration in the mutant cell. This implies that the same mutation may result in different phenotypes (i.e., gene expression profiles) depending on the APC content of the cell in which the mutation occurs. A prominent example is the expression profile of genes regulated by cooperative activation. While the mutation has a strong impact on the mRNA steady state concentration at 100 nM APC, there is almost no effect if this mutation occurs at 10 nM APC (Figure 9B). The analysis shows that at both APC concentrations considered here, the expression of genes regulated by a mass action mechanism is strongly susceptible to mutations. In contrast, the steady state expression of iFFL-regulated genes is not changed by the mutation at either APC concentrations.

In our study we simplified the mutation mechanisms and only considered their potential to modify the APC-dependent degradation of β-catenin. It might be speculated that a mutation in β-catenin not only affects its stability but also its transcriptional activity. In an experimental study, the expression of target genes in hepatocellular carcinoma harboring a mutation in either Axin or β-catenin has been measured (Zucman-Rossi et al., 2007). This study has revealed that gene expression might be different depending on whether Axin or β-catenin is mutated. These differences cannot be reproduced by our minimal model due to the strong simplification made for the molecular mechanisms involved in APC-dependent β-catenin degradation and the simplified implementation of the mutation.

In our modeling approach, we consider individual cells independently of their neighbors. However, some Wnt/β-catenin target genes encode for proteins which affect intercellular signaling, the gradient and/or its effectiveness. For instance, DKK1 is a Wnt/β-catenin target gene and its product influences Wnt/β-catenin signaling of the surrounding cells by competing with Wnt ligands for receptor binding (Mao et al., 2001; Chamorro et al., 2005). The expression and cell surface representation of Wnt receptors LRP and Frizzled is also regulated by Wnt/β-catenin signaling (Hao et al., 2012). Target gene products that modulate the effectiveness of the gradient are discussed to increase the stability against fluctuations in the gradient (Jaeger et al., 2008). Other theoretical investigations have shown that the exchange of molecules between cells in a gradient may produce even more complex spatio-temporal behavior (Schütze and Wolf, 2010).

Our minimal signaling model reproduces the β-catenin steady states and dynamics upon constant Wnt stimulation of the detailed model and in fact extends the detailed model with respect to the representation of β-catenin/TCF complex concentration ranges and the inclusion of gene regulatory mechanisms. The parameters and total concentrations of the minimal model are predominantly derived from the detailed model (Lee et al., 2003). There are no comprehensive experimental time series data sets available to estimate the kinetic parameters of mammalian cell types. Recent experiments on the quantification of pathway components in resting mammalian cells show that key components such as Axin, APC, and β-catenin may be expressed in a highly cell-type specific manner (Chen et al., 2010; Schwanhausser et al., 2011; Tan et al., 2012). To what extent variations in the absolute concentrations have consequences for the signaling dynamics and downstream gene expression remains to be investigated. Our minimal model with its extension toward target gene regulation provides a useful and versatile basis for future modeling approaches of the Wnt/β-catenin pathway.

## Materials and Methods

### Numerical simulations

The changes of the concentrations of the system’s components are described by a set of ODEs and algebraic conservation equations. The particular equations and parameters are listed in Section “Model equations and parameters” in the Appendix. All calculations are done with Mathematica 8.0 (Wolfram Research, Inc.). Steady state solutions are numerically obtained.

### Fitting of reporter data

Published experimental data of β-catenin reporter constructs (Biechele and Moon, 2008) are analyzed to gain insights into possible mechanisms of target gene activation. Three possible kinetics are considered: (i) linear mass action kinetics, (ii) Michaelis–Menten kinetics, and (iii) Hill-type activation. In 500 independent fitting runs each, the kinetics (i), (ii), and (iii) are fitted to the data using a log-likelihood approach. The AICc values are calculated to identify the kinetics which explains the data best. Details on the fitting procedure are provided in Section “Fitting of reporter data” in the Appendix.

## Conflict of Interest Statement

The authors declare that the research was conducted in the absence of any commercial or financial relationships that could be construed as a potential conflict of interest.

## Appendix

### Model equations and parameters

#### The signal transduction module of the minimal model

Figure 1 shows the schematic representation of the minimal model illustrating two different mechanisms of Wnt/β-catenin target gene regulation. In both schemes, the signal transduction module (highlighted with a gray background) is identical. It consists of reactions 1–9 and is given by the Eqs A1–A16. In the schemes, the number next to an arrow denotes the number of the particular reaction. Components in a complex are separated by a slash.

Differential equations

(A1) $$\begin{array}{ll} & \frac{\text{d}\left(\beta^{\text{\_}}\text{catenin}\right)}{\text{d}t}=v_{1}-v_{2}-v_{3}-v_{4}-v_{5} \end{array}$$

(A2) $$\begin{array}{ll} & \frac{\text{d}\left(\text{APC}\right)}{\text{d}t}=-v_{4} \end{array}$$

(A3) $$\begin{array}{l} \frac{\text{d}\left(\text{TCF}\right)}{\text{d}t}=-v_{5}+v_{6}-v_{7} \end{array}$$

(A4) $$\begin{array}{l} \frac{\text{d}\left(\beta^{\text{\_}}\text{catenin}∕\text{TCF}\right)}{\text{d}t}=v_{5} \end{array}$$

(A5) $$\begin{array}{l} \frac{\text{d}\left(\text{Ds}{\text{h}}_{a}\right)}{\text{d}t}=v_{8}-v_{9} \end{array}$$

Conservation relations

(A6) $$\begin{array}{ll} & \text{Ds}{\text{h}}^{\text{tot}}=\text{Ds}{\text{h}}_{i}+\text{Ds}{\text{h}}_{a} \end{array}$$

(A7) $$\begin{array}{ll} & \text{AP}{\text{C}}^{\text{tot}}=\text{APC}+\left(\beta^{\text{\_}}\text{catenin}∕\text{APC}\right) \end{array}$$

Rate equations

(A8) $$\begin{array}{ll} v_{1} & =\text{const}. \end{array}$$

(A9) $$\begin{array}{ll} v_{2} & =k_{2}\cdot \beta^{\text{\_}}\text{catenin} \end{array}$$

(A10) $$\begin{array}{ll} v_{3} & =\frac{k_{3}\cdot \text{APC}\cdot \beta^{\text{\_}}\text{catenin}}{K+\text{Ds}{\text{h}}_{a}} \end{array}$$

(A11) $$\begin{array}{ll} v_{4} & =k_{4}\cdot \text{APC}\cdot \beta^{\text{\_}}\text{catenin}-k_{-4}\cdot \left(\beta^{\text{\_}}\text{catenin}∕\text{APC}\right) \end{array}$$

(A12) $$\begin{array}{ll} v_{5} & =k_{5}\cdot \text{TCF}\cdot \beta^{\text{\_}}\text{catenin}-k_{-5}\cdot \left(\beta^{\text{\_}}\text{catenin}∕\text{TCF}\right) \end{array}$$

(A13) $$\begin{array}{ll} v_{6} & =\text{const}\text{.} \end{array}$$

(A14) $$\begin{array}{ll} v_{7} & =k_{7}\cdot \text{TCF} \end{array}$$

(A15) $$\begin{array}{ll} v_{8} & =k_{8}\cdot \text{Ds}{\text{h}}_{i}\cdot \text{Wnt} \end{array}$$

(A16) $$\begin{array}{ll} v_{9} & =k_{9}\cdot \text{Ds}{\text{h}}_{a} \end{array}$$

#### Regulation of the target gene expression by the iFFL motif

The effects of the iFFL motif (Figure 1B) are investigated in Section “Wnt/β-catenin target gene regulation by an incoherent feedforward loop motif”. For the analyses, the signal transduction module (Eqs A1–A16) is extended by the Eqs A17–A22.

Additional differential equations of the iFFL motif

(A17) $$\begin{array}{ll} & \frac{\text{d}\left(\text{repressor}\right)}{\text{d}t}=v_{12}-v_{13} \end{array}$$

(A18) $$\begin{array}{l} \frac{\text{d}\left(\text{mRNA}\right)}{\text{d}t}=v_{10}-v_{11} \end{array}$$

Additional rate equations of the iFFL motif

(A19) $$v_{10}=v_{10}^{\max}\cdot \frac{\frac{\left(\beta -\text{catenin}/\text{TCF}\right)}{K_{1}}}{1+\frac{\left(\beta -\text{catenin}/\text{TCF}\right)}{K_{1}}+\frac{\text{repressor}}{K_{2}}+\frac{\left(\beta -\text{catenin}/\text{TCF}\right)\cdot \text{repressor}}{K_{3}}}$$

(A20) $$v_{11}=k_{11}\cdot \text{mRNA}$$

(A21) $$v_{12}=k_{12}\cdot \left(\beta^{\text{\_}}\text{catenin}∕\text{TCF}\right)$$

(A22) $$v_{13}=k_{13}\cdot \text{repressor}$$

$v_{10}^{\max}$ is the maximal transcription rate, *K*_1_, *K*_2_, and *K*_3_ are effective binding constants.

#### Activation of the target gene expression

In Section “Activation of Wnt/β-catenin target genes”, the differences between linear and cooperative activation of the mRNA production by β-catenin/TCF complexes are analyzed. To this end, the signal transduction module is extended by either a linear or a Hill kinetics mRNA production term. All together, the model consists of Eqs A1–A16 (signal transduction module), Eq. A23 (ODE of the temporal change of the mRNA), Eq. A26 (degradation of the mRNA), and either Eq. A24 or Eq. A25 in the case of linear or Hill-type activation kinetics of the mRNA production, respectively.

Additional differential equations of the activation of the gene expression

(A23) $$\frac{\text{d}\left(\text{mRNA}\right)}{\text{d}t}=v_{10}-v_{11}$$

Additional rate equations of the activation of the gene expression

(A24) $$\begin{array}{lll} v_{10} & =k_{10}\cdot \left(\beta^{\text{\_}}\text{catenin}∕\text{TCF}\right) & \end{array}$$

(A25) $$v_{10}=v_{10}^{\max}\cdot \frac{{\left(\beta^{\text{\_}}\text{catenin}∕\text{TCF}\right)}^{n}}{K_{\text{M}}^{n}+{\left(\beta^{\text{\_}}\text{catenin}\text{/}\text{TCF}\right)}^{n}}+v_{10}^{\text{basal}}$$

(A26) $$v_{11}=k_{11}\cdot \text{mRNA}$$

$v_{10}^{\max}$ is the maximal inducible transcription rate, $v_{10}^{\text{basal}}$ is an additional basal transcription rate, *K*_M_ describes the β-catenin/TCF complex concentration at half maximal inducible transcription rate, and *n* is the Hill coefficient, which represents a measure of the cooperativity between the individual β-catenin/TCF complexes.We use a Hill coefficient of three throughout the manuscript unless stated otherwise. Note that for *n* = 1, Eq. A25 describes a Michaelis–Menten kinetics.

#### Inhibition of target gene expression by β-catenin/TCF complexes

The effects of repression of mRNA production by the β-catenin/TCF complex are investigated in Section “Repression of Wnt/β-catenin target genes”. For these analyses, the model consists of the Eqs A1–A16 (signal transduction module), Eq. A27 (ODE of the temporal change of the mRNA), Eq. A29 (mRNA degradation), and Eq. A28 which describes the inhibition of the mRNA production by the β-catenin/TCF complex.

Additional differential equations of the inhibition of the mRNA expression

(A27) $$\frac{\text{d}\left(\text{mRNA}\right)}{\text{d}t}=v_{10}-v_{11}$$

Additional rate equations of the inhibition of the mRNA expression

(A28) $$v_{10}=v_{10}^{\max}\cdot \frac{1}{1+\frac{\left(\beta -\text{catenin}/\text{TCF}\right)}{K_{i}}}$$

(A29) $$\begin{array}{ll} v_{11} & =k_{11}\cdot \text{mRNA} \end{array}$$

$v_{10}^{\max}$ is the maximal transcription rate; *K*_i_ is the inhibition constant.

**Table A1:** **Kinetic parameters of the signal transduction module**.

Parameter	Value
*v*_1_	0.423 nM min^−1^
*k*_2_	2.57 · 10^−4^ min^−1^
*k*_3_	3.08 · 10^−3^ min^−1^
*K*	18 nM
*k*_4_	10^5^ nM^−1^ min^−1^
*k*_−4_	1.2 · 10^8^ min^−1^
*k*_5_	0.0333 nM^−1^ min^−1^
*k*_−5_	1 min^−1^
*v*_6_	0.686 nM min^−1^
*k*_7_	0.084 min^−1^
*k*_8_	8 · 10^−3^ nM^−1^ min^−1^
*k*_9_	6.7 · 10^−4^ min^−1^

**Table A2:** **Total protein concentrations of proteins obeying a conservation relation**.

Component	Value
APC^tot^	100 nM
Dsh^tot^	100 nM

#### Transcriptional feedback via TCF

In the Sections “Wnt/β-catenin target gene regulation by an incoherent feedforward loop motif” to “Repression of Wnt/β-catenin target genes”, the consequences of the three different regulatory mechanisms are analyzed under the assumption of a constant production of TCF (Eq. A13). In Section “Transcriptional feedback regulation via TCF family members”, the effect of the different regulatory motifs is investigated in the presence of a transcriptional feedback via TCF. In this case, the TCF synthesis is not constant but depends on the mRNA level. For the analyses of transcriptional feedback effects, we thus replace the Eq. A13 by Eq. A30 in all cases.

(A30)
$$v_{6}=k_{6}\cdot \text{mRNA}$$

**Table A3:** **Kinetic parameters of the iFFL motif**.

Parameter	Value
$v_{10}^{\max}$	1368.18 nM min^–1^
*k*_11_	10^–2^ min^–1^
*k*_12_	4.44 · 10^–3^ min^–1^
*k*_13_	10^–3^ min^–1^
*K*_1_	1300 nM
*K*_2_	0.03 nM
*K*_3_	4 · 10^4^ nM

**Table A4:** **Kinetic parameters of the linear and Hill-type activation of the gene expression**.

Parameter	Value
$v_{10}^{\max}$	0.03 nM min^–1^
$v_{10}^{\text{basal}}$	6.1 · 10^–3^ nM min^–1^
*n*	3
*k*_10_	0.03 min^–1^
*k*_11_	10^–2^ min^–1^
*K*_M_	23 nM

**Table A5:** **Additional kinetic parameters of the repression motif**.

Parameter	Value
$v_{10}^{\max}$	8.84 · 10^–3^ nM min^–1^
*k*_11_	10^–2^ min^–1^
*K*_i_	23 nM

**Table A6:** **Kinetic parameter of the TCF feedback**.

Parameter	Value
*k*_6_	1 min^–1^

### Fitting of reporter data

β-Catenin/TCF reporter experiments have been published in which the response of reporter constructs with 3, 8, and 12 TBEs to different Wnt concentrations has been compared (Biechele and Moon, 2008). The data that has been the basis of Figure 8.1.A in Biechele and Moon (2008) has been kindly provided. It tables the Wnt concentration as “percentage of Wnt conditioned medium” with the corresponding mean and standard deviation of three normalized reporter readout replicates.

To test for possible cooperativity between β-catenin/TCF complexes in the regulation of reporter expression, we first calculated the β-catenin/TCF complex concentrations for the given “percentage of Wnt conditioned medium” using our signal transduction module (Eqs A1–A16). For the calculations, we assumed that the “percentage of Wnt conditioned medium” equals the respective percentage of the simulated Wnt concentration of 1 nM. Moreover, following the basic assumption in reporter experiments, we consider the fold change of the reporter readout, i.e., Wnt-stimulated to control measurement, to be linearly correlated to the fold change of the reporter mRNA. The reporter mRNA concentration change over time can be described by the following ODE:

(A31) $$\frac{\text{d}\left(\text{mRNA}\right)}{\text{d}t}=v_{\text{induced}}+v_{\text{basal}}-k_{\text{degradation}}\cdot \text{mRNA}$$

in which *v*_induced_ denotes the β-catenin/TCF-complex-dependent transcriptional activation rate, *v*_basal_ is a basal transcription rate, and *k*_degradation_ denotes the degradation rate constant in the linear reporter mRNA degradation rate. As the reporter readout is measured at steady state condition, we derive the mRNA steady state concentration (mRNA_stst_) from Eq. A31:

(A32) $$\text{mRN}{\text{A}}_{\text{stst}}=\frac{v_{\text{induced}}+v_{\text{basal}}}{k_{\text{degradation}}}$$

**Table A7:** **Corrected Akaike information criterion of the best fits**.

Number of TBEs	Linear kinetics	Michaelis–Menten kinetics	Hill-type kinetics
3	159.5	165.1	29.0
8	558.7	564.3	51.9
12^a^	7021.6	7027.2	66.1
12^b^	16728.3	16733.9	328.0

*For all three kinetics, (i) linear mass action kinetics, (ii) Michaelis–Menten kinetics, and (iii) Hill-type activation, the calculated values of the corrected Akaike information criterion are given for the four data sets*.

*^a^Transiently transfected reporter construct with 12 TBEs; ^b^stable integrated reporter construct with 12 TBEs*.

In the absence of a Wnt stimulus, i.e., *v*_induced_ = 0, this term simplifies to the basal reporter mRNA steady state (mRNA_basal_). Since the experimental data shows a standard deviation in the control data point, we take a small error ε in the basal reporter mRNA steady state into account.

(A33)
$$\text{mRN}{\text{A}}_{\text{basal}}=\frac{v_{\text{basal}}\pm \varepsilon }{k_{\text{degradation}}}$$

The experiments report the fold change of the reporter mRNA, which gives the ratio of the mRNA steady state to the basal mRNA steady state. According to Eqs A32 and A33, the fold change is given by:

(A34) $$\text{fold}\;\text{change}=a\cdot v_{\text{induced}}+c$$

where $a=\frac{1}{v_{\text{basal}}\pm \varepsilon }$ is a scaling factor and $c=\frac{v_{\text{basal}}}{v_{\text{basal}}\pm \varepsilon }$ is the offset constant along the ordinate.

Next, we considered three possible transcriptional activation kinetics in *v*_induced_ in Eq. A34: (i) linear mass action kinetics, (ii) Michaelis–Menten kinetics, and (iii) Hill-type activation:

(A35) $$\begin{array}{ll} \left(\text{i}\right)\;\text{fold}\;\text{change} & =a\cdot \left(\beta^{\text{\_}}\text{catenin}\text{/}\text{TCF}-x_{0}\right)+c \end{array}$$

(A36) $$(\text{ii})\text{ fold change}=a\cdot \frac{\left(\beta -\text{catenin}/\text{TCF}-x_{0}\right)}{K_{M}+\left(\beta -\text{catenin}/\text{TCF}-x_{0}\right)}+c$$

(A37) $$(\text{iii})\text{ fold change}=a\cdot \frac{{\left(\beta -\text{catenin}/\text{TCF}-x_{0}\right)}^{n}}{K_{M}^{n}+{\left(\beta -\text{catenin}/\text{TCF}-x_{0}\right)}^{n}}+c$$

where *a* is the scaling constant, *x*_0_ and *c* are constants accounting for an offset along the abscissa and ordinate, respectively. *K*_M_ describes the β-catenin/TCF complex concentration at half maximal transcriptional activation rate and the Hill coefficient *n* represents a measure of the cooperativity between the individual β-catenin/TCF complexes.

We fitted the functions (i), (ii), and (iii) to the data set of each reporter construct. In the fitting procedure the log-likelihood function, that is the weighted sum of squares of the residuals between the data and the simulations of the activation kinetics, was minimized. Each residual was weighted with respect to the corresponding standard deviation of the data point. We performed 500 independent fitting runs choosing different starting parameter values for each construct to obtain the global minimum of the log-likelihood function. Considering the best fit for each function we compared the calculated values of the AICc to identify the kinetics which explains the data best (Hurvich and Tsai, 1995; Lukacs et al., 2010). The AICc was calculated by:

(A38) $$\text{AICc}=2\cdot p-2\cdot \ln\left[L\right]+\frac{2\cdot p\cdot \left(p+1\right)}{d-p-1}$$

where *p* is the number of parameters of the tested kinetics, *d* denotes the sample size (i.e., *d* = 8 data points), and ln[*L*] is the log-likelihood function. Our calculations show that the Hill-type activation kinetics (iii) yields the smallest AICc value and thus approximates the data best (Figure 5A; Figure A2) suggesting a cooperative regulation of the reporter expression.

Next, the group of best fits, i.e., those whose log-likelihood function value deviated at most 20% from the minimal value of the log-likelihood function, is used to gain information on the variability of the Hill coefficients. Their analysis revealed that the Hill coefficients *n* are all higher than 1 (Figure 5B). The values of the Hill coefficients span from 2.0 to 4.2 for the 3 TBEs construct. For the 8 TBEs and the two different 12 TBEs constructs, the Hill coefficients take values in a narrower range between 3.9 and 4.2 with medians of 3.9, 4.0, and 3.9, respectively. In contrast, the median of the construct with the 3 TBEs is lower (2.6) than that of the other three constructs. This may point to a limitation of the number of β-catenin/TCF complexes that can act cooperatively in the regulation of the reporter constructs.

## Abbreviations

AICc, corrected Akaike information criterion; APC, adenomatous polyposis coli; Arg1, arginase 1; Cps1, carbamoyl-phosphatase 1; DKK, Dickkopf; Dsh, Dishevelled; Gls2, glutaminase 2; Glt1, glutamate transporter 1; GS, glutamine synthetase; iFFL, incoherent feedforward loop; Lect2, leukocyte cell-derived chemotaxin 2; LEF, lymphoid enhancer-binding factor; OAT, ornithine aminotransferase; ODE, ordinary differential equation; TBE, TCF/LEF binding elements; TCF, T-cell factor.

## Research Topic:

From structural to molecular systems biology: experimental and computational approaches to unravel mechanisms of kinase activity regulation in cancer and neurodegeneration

## Topic Editor:

Matteo Barberis, Humboldt University Berlin, Germany; Max Planck Institute for Molecular Genetics, Berlin, Germany
